# The Use of the Diode Laser against the Microbiome on Composites Closing the Screw Access Hall (Sah) in the Reconstruction of Dental Implants: Ex Vivo Studies

**DOI:** 10.3390/ijerph19127494

**Published:** 2022-06-18

**Authors:** Anna Wawrzyk, Mansur Rahnama, Weronika Sofińska-Chmiel, Sławomir Wilczyński, Michał Łobacz

**Affiliations:** 1 Silesian Park of Medical Technology Kardio-Med Silesia in Zabrze, M. Curie Skłodowskiej 10C Str., 41-800 Zabrze, Poland; anna.wawrzyk@gazeta.pl; 2 The Chair and Department of Oral Surgery, Medical University of Lublin, Chodźki 6, 20-093 Lublin, Poland; rahnama.m@interia.pl; 3 Analytical Laboratory, Institute of Chemical Sciences, Faculty of Chemistry, Maria Curie Skłodowska University, Maria Curie Skłodowska Sq. 2, 20-031 Lublin, Poland; weronika.sofinska-chmiel@mail.umcs.pl; 4 Department of Basic Biomedical Science, Faculty of Pharmaceutical Sciences in Sosnowiec, Medical University of Silesia, Kasztanowa 3, 41-205 Sosnowiec, Poland; swilczynski@sum.edu.pl

**Keywords:** composite, diode laser, microorganisms, dental implant, dental material, screw access hole

## Abstract

Patients undergoing implant treatment are at risk of peri-implant bone loss, which is most often caused by the adverse effects of microorganisms, but there are few proven procedures for their reduction. The aim of the research was to identify the microorganisms inhabiting the composites used to close the screw access hole (SAH), compare them numerically with those present on the surface of crowns and teeth, and optimize the doses of the diode laser, which will reduce microorganisms and will not deteriorate the roughness of polished composites. Patients were swabbed from the surface of SAH composites, from porcelain and zirconium restorations, and from teeth, and then the number of microorganisms was determined by using a culture technique. Microorganisms were identified by MALDI–TOF MS and NGS sequencing. The effectiveness of diode laser irradiation was achieved by using four variants of exposure. After polishing and laser irradiation, the surface roughness of the composites was studied by using optical profilometry. On the surface of SAH, 10^6^ to 10^8^ microorganisms were identified at 0.4 cm^2^, including many pathogenic species. Among the materials used for the reconstruction of dental implants, the greatest microbiological contamination was found on the composites used to close the SAH. The diode laser with a wavelength of 810 nm with an average power of 3.84 W, during 60 s and 2 × 30 s, has a biocidal effect and does not significantly change the surface roughness of composites. The best reduction of microorganisms was achieved on a composite polished with a polishing rubber and then with a Sof-Lex™ Pre-Polishing Spiral beige (3M ESPE, Ave. St. Paul., MN, USA). Studies have shown that using the optimal laser dose can help treat periimplantitis. These studies provide important information on the possibility of the effective elimination of microorganisms by using a diode laser in the treatment of peri-implant bone loss.

## 1. Introduction

The success of implantoprosthetic treatment depends on many factors, including the correct implementation of the implant procedure, the correct laboratory fabrication of the crown, and its attachment to the implant, as well as the method of the composite surface preparation. All activities, especially those aimed at the proper preparation and maintenance of microbiological cleanliness on the surfaces of materials used in implantoprosthetic treatment contribute to minimizing the risk of complications. One of the main etiological factors of this disease are microorganisms, including pathogens which are deposited on largely susceptible surfaces of dental materials. Other factors that are mentioned in the literature that may affect the development of peri-implant inflammations are also cracks in the abutment screw or the method of fixing the prosthetic crown on the implant [1,2]. Oral microorganisms are most often in the form of a biofilm. Oral biofilm diseases, including those surrounding implants are exceptional infections, because they develop from resident native microflora [3]. To survive, microorganisms must adhere to soft or hard tissues. Replacing the mucosa epithelium three times a day is an effective defense mechanism, as it prevents the accumulation of large masses of microorganisms. However, surfaces that do not exfoliate, such as teeth, crowns, or endosseous implants, allow the formation of thick biofilms.

During the implant–prosthetic procedures the composition of the microbiome changes and microorganisms find their way into places that are usually inaccessible to them. The uncontrolled infection of microorganisms is mentioned as the main cause of implant failure [4]. The surfaces of the implant–prosthetic structure are contaminated with microorganisms to a varying degree. In their in vivo study, Cosyn et al. [5] proved significant contamination of SAH and the internal components of the implant, as well as the superstructure. Smith et al. [6] showed that bacteria accumulate in the micro-fissure at the implant–abutment interface and cause a marginal bone loss. Mahony et al. [7] found out that the accumulation of plaque occurs along the implant–abutment, abutment–crown, and implant–crown and on the abutment, implant, and crown surfaces. The formation of biofilm is facilitated by the increase in roughness of implant–prosthetic surfaces [8,9]. Teughels et al. showed that, already above R = 0.2 µm, the surface roughness predisposes the filling materials to the deposition of a greater number of biofilm-forming microorganisms [9]. The surface roughness can increase for various reasons. According to Nedeljkovic et al. [10], composites can biodegrade in the oral cavity depending on their composition. Moreover, *Streptococcus mutans* increases the roughness of tested composites by 12–44%. There are many other factors that affect the decomposition time of composites. Currently, more attention is paid to improving the materials resistance of to the biofilm degradation in the oral cavity environment caused by aqueous solvents and salivary enzymes, as well as to the biofilm development [11]. In order to increase the durability of composite dental restorations, various methods of producing composites are sought, taking into account the factors responsible for degradation [12].

Natural teeth differ in roughness from the materials of which the crowns are made, i.e., porcelain, zirconium, or composite. The roughness of teeth and crowns after application is usually not modified, but that of the composite depends on the polishing procedure used by the dentist. Proper composite polishing can minimize the degree of microbial deposition and, thus, the formation of unfavorable biofilms.

One of the final stages of the implant–prosthetic procedure is the SAH closure. Therefore, it is essential for the surfaces of the complementary composites to possess properties favoring the adhesion of biofilms as little as possible. An improperly prepared and poorly smoothed composite can become a reservoir of microorganisms and, through the degraded surface, can contribute to the microorganisms’ penetration to the inner part of the SAH.

Composites have become one of the most widely used aesthetic restorative materials. The composite used traditionally in dentistry contain large particles of ground amorphous silica and quartz and, therefore, have good mechanical properties. However, this makes the surface increasingly rougher with daily abrasion. In addition, a damage is observed at the interface between the composite and other surfaces [13].

Using various composite polishing procedures by dentists, the probability of the microorganisms’ deposition on the surfaces of the implant–prosthetic structures and the permeability of gaps can be reduced or increased. Finishing and polishing procedures affect the surface quality of the composite [14,15]. The rough surface contributes to the accumulation of plaque, which favors the development of oral diseases [16,17].

The systematic reduction of the number of microorganisms on composites surface is of great importance. Proper oral hygiene has the greatest impact, but it is not effective in all cases. Therefore, attempts are made to decontaminate the composite by using various methods. Cavalcanti et al. [18] attempted to reduce the presence of microbes in SAH, but microbial infiltration did occur. Antiseptic materials were also tested to seal the micro-gap; when applied, they reduced the risk of contamination and improved the condition of soft tissues [19]. One of the methods that can reduce the number of microorganisms on the composites’ surface is the use of a diode laser, the irradiation of which can supplement the implantological treatment procedure. The effectiveness of the diode laser against microorganisms inhabiting porcelain, zircon, and titanium was confirmed by Wawrzyk et al. [20,21,22]. The method of decontamination with the diode laser used by Wawrzyk et.al. did not adversely affect the surface structure of the materials.

The range of hard intraoral surfaces’ roughness is wide, and the effectiveness and safety of dental treatments depend upon the procedures used and the type of materials.

Some techniques result in a very sleek surface, while others result in the surface being rougher. In order to obtain and keep the material surface as smooth as possible, polishing is used [17]. Polishing kits can have a different effect on the composite surfaces. Kocaagaoglu et al. found no statistically significant difference between the composites in terms of surface roughness after polishing [23]. Sarac et al. obtained the highest Ra (roughness factor—the lower the value, the smoother the surface) values for the hybrid composite resins due to the large size of the filler particles exposed after polishing. He obtained the smoothest surfaces with the use of polyester strips, and the use of glaze after polishing pads resulted in significantly smaller Ra values than using the discs alone, confirming that the glaze appears to fill structural microdefects and provide a more uniform and regular surface [24]. Yasser et al. showed that, by using the one-step PoGo polishing system, a much smaller surface roughness is achieved compared to the Sof-Lex polishing system [25].

The aim of the study was to present the microbiological contamination of composites used to close the screw access opening constituting the prosthetic reconstruction of dental implants and a quantitative comparison of the SAH microbiome and crowns. For comparison, tests of a natural tooth were also performed. In addition, an attempt was made to optimize the operation of the diode laser during the disinfection irradiation of polished composites by using three methods, with the simultaneous assessment of the surface structure after these treatments.

## 2. Materials and Methods

### 2.1. Patients and Materials

**Figure 1:**
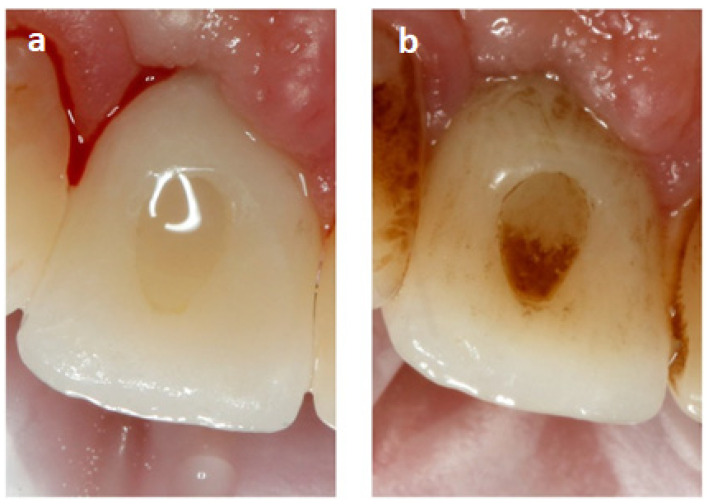
Intraoral photo showing the all-ceramic crown on the implant in the tooth position 11 after the professional oral cleaning (**a**) and 1.5 years later (**b**). Palatine surface view.

In order to confirm that the composites used for closing SAH within a porcelain implant crown accumulate impurities, photos were taken of a patient who was a tobacco smoker and did not care for oral hygiene properly (Figure 1).

Among the many patients undergoing implantoprosthetic treatment, the subjects were selected for the tests who gave their consent and had two zirconium and two porcelain crowns at the same time, so that the environmental conditions of the oral cavity were maintained, e.g., surrounding saliva. This was the main limitation of the sample size. Swabs for metagenomic and microbiological tests were taken from the surface of composites used for closing SAH on the crown of zirconium and porcelain from 6 patients of various ages: Patient 1—50 years, Patient 2—62 years, Patient 3—76 years, Patient 4—42 years, Patient 5—44 years, and Patient 6—35 years. Their implants were in the following positions: Patient 1, positions 46 (porcelain) and 36 (zirconium); Patient 2, positions 46 (porcelain) and 36 (zirconium); Patient 3, positions 16 (porcelain) and 26 (zirconium); Patient 4, positions 25.26 (porcelain) and 15.16 (zircon); Patient 5, positions 36.37 (porcelain) and 46.47 (zircon); and Patient 6, positions 15.16 (porcelain), 25.26 (zircon).

In each patient, SAHs were closed by using a 3M ESPE FiltekTM Z250 composite polished with the Sof-Lex system with final smoothing. Microbiological contamination of the adjacent healthy teeth, as well as the surface of the superstructure made of zirconium and porcelain, was examined for comparison.

The API (Approximal Plaque Index) ranged from 47 to 69% in the examined patients, indicating that they did not maintain proper oral hygiene. PPD (Pocket Probing Dept) was <4, which means that there was no visible periimplantitis. BoP (Bleeding on Probing) was positive around the tested implants; thus, bleeding was present on probing.

### 2.2. Methods

#### 2.2.1. Composite Visualization

To visualize impurities, photos of the crown of the dental implant were taken, along with the composite used to seal the SAH. Photos were taken with a Canon EOS 750D camera, Canon 100 mm f/2.8 L EF Macro IS USM lens, exposure time of 1/200, f/25 shutter, 100 mm lens focal length, and recorded in the CR3 format with 6000 × 4000 px resolution.

#### 2.2.2. Assessment of Microbiological Contamination of SAH

In order to compare the number of microorganisms inhabiting the surfaces of composites with the number of microorganisms present on the adjacent surfaces, swabs were taken from the surfaces of 0.4 cm^2^ of the composite, zirconium, porcelain, and teeth of 3 patients with the composite fillings in both zirconium and porcelain crowns.

The swab was placed in the sterile saline, shaken, and the initial suspension was prepared. The quantitative microbiological assessment was performed by using a spread plate technique (deep inoculation) by inoculating 0.3 mL from a 10-fold diluted sample to a TSA Tryptic Soy Agar medium plate three times. After the incubation at 36 ± 2 °C for 48 h, the colony-forming units (CFUs) were counted, and an average of three counts was calculated. The result is given in CFU/0.4 cm^2^.

For the microorganism identification, 0.3 mL of suspension (surface culture) was inoculated onto a Columbia Blood Agar medium. Plates were incubated at 36 ± 2 °C for 48 h under the aerobic conditions with 5% CO_2_, and each of the cultured colonies was transferred into the TSA agar for multiplication.

Each colony was subsequently identified by using the matrix-assisted laser desorption ionization–time-of-flight mass spectrometry (MALDI–TOF MS) method based on the Microflex LT system (Bruker Daltonics, Bremen, Germany), the IVD HCCA matrix (Bruker, Billerica, MA, USA).

Identification of the selected microorganisms’ species was confirmed by using the 16sRNA technique based on the comparison in the BLAST program (http://blast.ncbi.nlm.nih.gov) Thu, 17 March 2022 of the obtained DNA sequence of the ITS (Internal Transcribed Spacer) region of the sample. The sequences were analyzed in CLC Main Workbench 8 and examined by using the BLAST software.

The identification of microorganisms was extended to include the metagenomic analysis by the next-generation sequencing (NGS) of the V3/V4 region of the 16S rRNA gene and the ITS1 region, using the MiSeq instrument (Illumina, San Diego, CA, USA) to determine DNA of all microbial species present in the swab samples taken from the composite surfaces but that could not be grown under laboratory conditions.

#### 2.2.3. Polishing of the Model Composites

Model plates made by a dentist from 3M ESPE FiltekTM Z250 composite (Universal Restorative A2Shade Ref 6020A2 Made in USA by 3M ESPE Dental Products 2510 Conway Ave. St. Paul. MN55144-1000 USA. LOT NC 94679, expiry date 30 January 2024), were used to assess surface roughness after applying 3 different polishing techniques, also before and after application of a laser. The composite was smoothed in the same way as the patients’ composites, using the same techniques, tools, and polishing time.

The Sof-Lex™ directional polishing system was used to smooth the composite tile dental models. The system without water cooling was used for 30 s by applying a dental contra-angle handpiece and a rotational speed of 15,000 rpm. A one-step directional polishing system with the cone-shaped Enhance (Dentsply Sirona, Charlotte, North Carolina, USA) polish rubber cup was used for the K1 composite; a two-step system using rubber, followed by the Sof-Lex™ Pre-Polishing Spiral (beige) (3M™) polishing disc, which, according to the manufacturer’s declarations should remove scratches from the K2 composite fillings; the three-step polishing system composed of polish rubber, Sof-Lex™ Pre-Polishing Spiral (beige) (3M™), ended with the Sof-Lex™ Pre-Polishing Spiral (pink) (3M™) polishing disc being used to provide gloss for the K3 composite. The pink rubberized polishing disc is impregnated with diamond particles.

In the case of two- and three-step polishing, the surface of the composite was rinsed with water for 10 s after using each tool and dried for 10 s, using a dental air syringe blower before another polishing tool was applied.

#### 2.2.4. Laser Irradiation

##### Irradiation of Polished Model Composites for Roughness Testing

The samples were irradiated with the Elexxion claros (AG, Singen, Germany) laser with the fiber diameter of 600 μm at the wavelength of λ = 810 ± 10 nm according to the L3 variant periimplantitis surgical: 25 W/15,000 Hz/10 μs, mean = 3.84 W, tip 600 μm for 60 s and, additionally, for 2 × 30 s with 60 s cooling, using the sweeping technique. In addition, the composites were irradiated in one place with the L3 variant for 60 s, using the contactless point technique.

#### 2.2.5. Assessment of Decontamination Effectiveness on the Polished Composites

The samples were irradiated with the Elexxion claros (AG, Singen, Germany) laser with the fiber diameter of 600 μm, at the wavelength of λ = 810 ± 10 nm, in four variants. L1—decontaminate implant: 1 W/CW, mean = 1.0 W, tip 600 μm, t = 60 s. L2—expose implant: 15 W/15,000 Hz/10 μs, mean = 2.30 W, tip 600 μm, t = 60 s. L3—periimplantitis surgical: 25 W/15,000 Hz/10 μs, mean = 3.84 W, tip 600 μm, t = 60 s; moreover, L3 over time t = 2 × 30 s with 60 s cooling between exposures (not recommended by the manufacturer). The contactless surface lasering (sweeping) technique was used for all samples.

To assess the biocidal efficacy of the diode laser, microorganism species were identified by using MALDI–TOF MS. The most common and potentially pathogenic microorganisms aerobic were selected for the study: *Klebsiella oxytoca*, *Rothia dentocariosa*, and *Streptococcus pneumoniae*, as well as the yeast-derived fungus *Candida guillermondi*. The inoculum at the concentrations of 10^7^ CFU/mL from the individual microorganisms isolated from the surface of composites was applied onto the composite model plates, using the densitometric method. As much as 50 μL was applied for each composite, waiting after each portion until it dried. These maneuvers were performed under a hood with the laminar vertical airflow. Subsequently, the plates were irradiated in three different laser variants, rinsing plates, making the initial suspension, and then, after preparing 10-fold dilutions, growing cultures in accordance with the methodology described in Section 2.2.2, using Blood Agar and incubating at 36 ± 2 °C for 48 h. The microbial reduction efficacy (R%) was calculated according to the following equation: R% = ((N0 − N)/N0) × 100%, where N0 is the number of microorganisms before exposure, and N is the number of microorganisms after exposure.

#### 2.2.6. Statistical Analysis

For the number of microorganisms inhabiting individual surfaces, the average value and the standard deviation were calculated. The one-way analysis of variance (ANOVA) and the least significant difference test (significance level *p* < 0.05) were used to assess the significance of the differences between the number of microorganisms on the samples that were subjected to laser irradiation and the non-irradiated ones. Statistical analyses were performed by using the Statistica 6.0 software (Statsoft, St Tulsa, OK, USA).

#### 2.2.7. Surface Morphology Analysis

##### Optical Profilometry

The profilometric tests of the composite surfaces were carried out with the use of the Contour GT-K1 optical profilometer by Veeco and the VSI technique. The roughness parameters were determined for three composites: K1, K2, and K3. The surface of each composite was prepared by using different polishing procedures.

For each composite, the roughness parameters were determined for 5 areas of the tested sample for the following scan size: 946 µm × 1261 µm. For proper determination of the roughness parameters, the sample slope was corrected, and the surface wariness was taken into account. The surface-roughness measurement results are presented with the expanded uncertainty (factor k = 2). The roughness measurement uncertainty, Ra, was estimated by taking into account the repeatability, recovery, de-calibration of the apparatus, and standard uncertainty. The uncertainty was estimated for the two extreme points of the Ra measurement range (upper and lower limits), with the assumption of a trapezoidal distribution.

Roughness tests were also carried out for the composites after the laser process. The samples were irradiated with the Elexxion claros (AG, Singen, Germany) laser, with a fiber diameter of 600 µm, at the wavelength of λ = 810 ± 10 nm, in the three variants: L3a (60 s sweeping), L3b (2 × 30 s sweeping), and L3c (60s without any movements). For each laser variant, 3 measurements were made for 3 selected laser areas. The roughness parameters and microgeometry maps were determined for the following scan size: 946 µm × 1261 µm.

## 3. Results

### 3.1. Visualization of the Place Where the Composite Was Used to Fill SAH before and after Sandblasting

The composite used to fill SAH accumulates a lot of impurities. Figure 1a shows an all-ceramic crown constituting the suprastructure of the dental implant in position 11 after cleaning and polishing the composite. The composite surface is clean, and there are no visible differences resulting from accumulation of deposits, including plaque. In Figure 1b, the picture made 1.5 years later shows the sediments covering the palatal surface of the crown. The composite is covered with the dark sediment formed as an effect of improper oral hygiene, tobacco smoke, and tannins in the food. A thick plaque is also formed on the dentin, constituting a reservoir of microorganisms.

### 3.2. Metagenome on the Composites Surface

**Figure 2:**
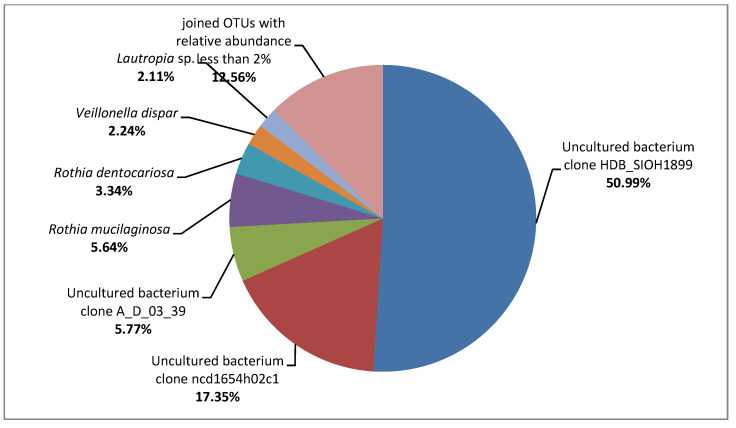
Identification results of the bacteria isolated from the composite surfaces, using the NGS method.

**Figure 3:**
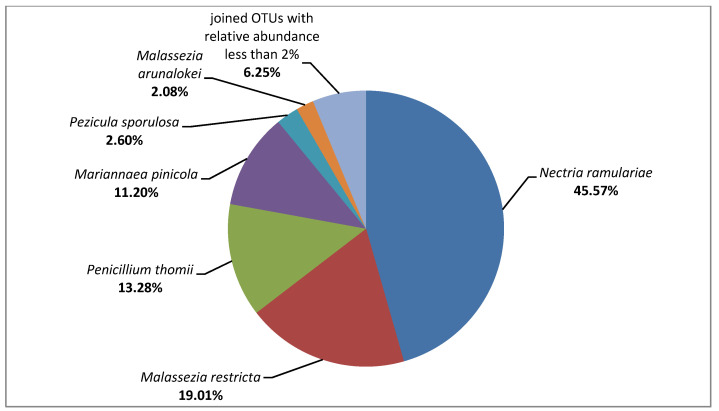
Identification results of the fungi isolated from the composite surfaces, using the NGS method.

After the NGS sequencing, 138,968 pairs of raw 16S rRNA gene sequence reads and 156,420 pairs of raw ITS1 region sequence reads were obtained, of which 69.24% and 22.87% were classified. For the tested composites, 96,166 reads were assigned to the kingdom of Bacteria, and only 384 reads to the Fungi kingdom. Due to the high level of contamination of the tested sample, mostly with genetic material of unknown (90.68%) and plant (7.68%) origin, the results of ITS1 analysis were additionally filtered to allow further analysis of only those taxonomic units which belong to the kingdom of Fungi. On the surfaces of tested composites, bacteria belonging to the Gammaproteobacteria class and fungi of the Sordariomycetes class were predominant (51.49% and 56.77%, respectively). The metagenomic analysis allowed us to identify bacteria belonging to the following families: Enterobacteriaceae (relative abundance: 51.00%), Streptococcaceae (17.46%), Micrococcaceae (9.23%), Leptotrichiaceae (5.77%), Actinomycetaceae (2.85%), Veillonellaceae (2.46%), Neisseriaceae (2.20%), and Burkholderiaceae (2.11%). Additionally, the composites were populated by the fungi belonging to the following families: Nectriaceae (56.77%), Malasseziaceae (21.09%), Aspergillaceae (13.28%), Dermateaceae (2.60%), and Cladosporiaceae (2.34%). All bacterial and fungal OTUs detected at the species level are shown in Figure 2 and Figure 3. This group includes uncultured bacterial clones belonging to the family of Enterobacteriaceae and genera of Streptococcus and Leptotrichia, for which 100% similarity at 100% of the sequence length to the strains described under the following GenBank accession numbers was demonstrated: HM185888.1, JF146348.1, and FJ470495.1, respectively.

Taking into account OTUs with the relative abundance <2%, a much greater variety of bacterial taxa than the fungal ones was detected at all analyzed taxonomic levels (e.g., 33 bacterial OTUs vs. 9 fungal OTUs at the family level, and 61 bacterial OTUs vs. 12 fungal OTUs at the species level). The following exemplary OTUs with the relative abundance <2% were detected for the bacteria Actinomyces sp., *Aggregatibacter segnis*, *Corynebacterium durum*, *Haemophilus parainfluenzae*, *Neisseria subflava*, Prevotella spp. (*P. melaninogenica* and *P. nigrescens), Rothia aeria*, and *Streptococcus anginosus*; and for the fungi *Alternaria alternata*, *Cladosporium delicatulum*, *Cladosporium halotolerans*, *Naganishia cerealis*, and *Saccharomyces cerevisiae.* Additionally, many of the sequences with the relative abundance <2% did not match to the cultivable microorganisms. For example, the bacteria belonging to the TM7 phylum (Saccharibacteria) were detected.

### 3.3. Quantitative and Qualitative Analysis of Microbial Contamination of Composites, Porcelain, Zirconium and Teeth Surfaces

Microbiological contamination of all tested surfaces in the patients ranged from 10^3^ to 10^8^ depending on the type of surface. On the composites, contamination ranged from 10^6^ to 10^8^. The largest number of microbes on all surfaces was detected in Patient 1. Among all surfaces, the smallest number of microorganisms was detected on teeth. In all patients, the number of microorganisms in the composite was the largest compared to the other surfaces (Table 1). The differences between the number of microorganisms on the teeth vs. the individual surfaces averaged two orders of magnitude in Patients 1 and 2, and in Patient 3, the difference amounted to three orders of magnitude. The difference between the number of microorganisms on the composite vs. zirconium is smaller than that between the composite and porcelain. In Patients 4, 5, and 6, the difference in the number of microorganisms between the surface of the teeth is one order of magnitude.

Nine dominant bacterial species and one species of fungus were isolated from the examined surfaces and identified by using the MALDI–TOF MS technique. They accounted for more than 90% of the grown microbes. Among them, *Streptococcus pneumoniae* accounted for 60% of all identified microbes, *Rothia dentocariosa* accounted for 25%, and the remaining 5% constituted *Rothia aeria* and *Neisseria subflava*.

In 39 cases out of 40, successively more microorganisms were detected on teeth, porcelain, and zirconium, and the most were on the composite (Table 2). The only exception is the case of the number of *Neisseria subflava* species, as more CFUs of this microbe were detected on zirconium in Patient 1 than on the composite. The least numerous was *Neisseria fluorescens* on zirconium in Patient 2, and the most numerous was *Streptococcus pneumoniae* in Patient 1. In Patients 1 and 2, four species of bacteria and one species of fungus were detected on the examined surfaces, whereas five species of bacteria and one fungus were identified in Patient 3. *Candida guillermondi* was identified on the suprastructures of the implants, but it was not present on the teeth in Patient 3 and tooth and porcelain in Patients 4, 5, and 6. This microorganism was also not found on zircons in Patient 5. *Klebsiella oxytoca* was detected on all surfaces in Patients 3 and 5. As the only ones, we detected *Neisseria flavescens* on zirconium and composite in Patient 2, *Neisseria perflava* on zirconium and composite in Patient 3, and *N. subflava* on both types of suprastructure and composite. *R. aeria* was not present in Patient 3, while in Patients 1 and 2, it colonized all surfaces. Similarly, *R. dentocariosa* was not identified only in Patient 2. Patient 1 did not grow *S. epidermidis* and *S. parasanguinis*. *S. pneumoniae* occurred on every surface in Patient 1 but it was not found in the other patients.

An additional identification was made confirming the affiliation of selected strains with the species. The DNA sequence of the 16S rRNA region is 100% identical to the *Rothia dentocariosa* sequence LR134479.1, 99% to the *Streptococcus oralis* sequence MH930451.1, 73% to the *Streptococcus pneumoniae* sequence MF578778.1., and 100% identical to the MK394108.1 sequence in *Meyerozyma guilliermondii*.

### 3.4. Surface Morphology Analysis

#### Composite Roughness Tests

**Figure 4:**
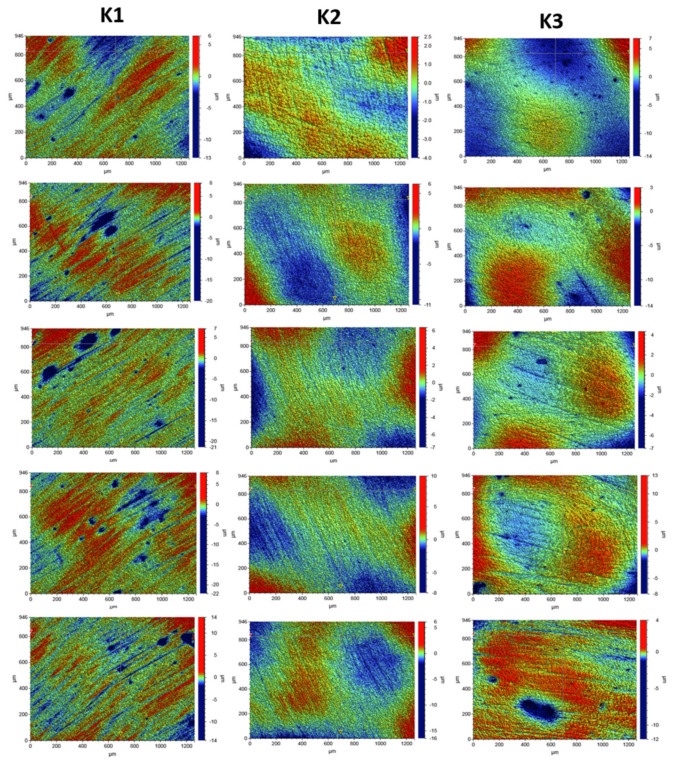
Surface microgeometry maps determined for the polished composites K1 (Enhance polishing pad), K2 (polishing pad, Sof-Lex™ Pre-Polishing Spiral (beige)), and K3 (polishing pad, beige polishing pad, pink polishing pad) before the laser irradiation process.

In order to assess the surface of K1, K2, and K3 composites smoothed in three different variants with an eraser and polishing wheels, five maps of surface microgeometry were made. Roughness parameters were also determined for these areas. The test results are shown in Figure 4. Table 3 shows the roughness parameters determined for the scanned area.

**Figure 5:**
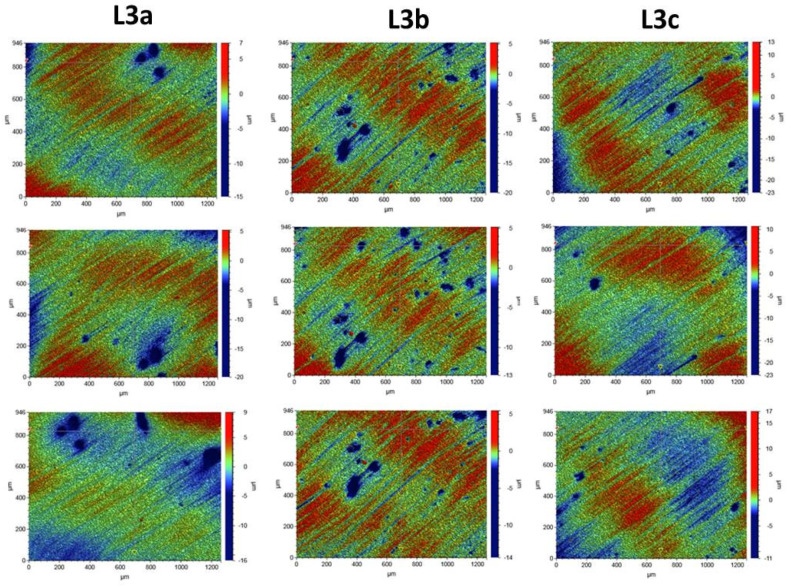
Surface microgeometry maps determined for the K1 composite for 3 variants of laser L3. L3a (periimplantitis variant, sweeping technique, 60 s), L3b (periimplantitis variant, sweeping technique, 2 × 30 s, with 60 s cooling break), and L3c (periimplantitis variant, scoring technique, 60 s).

**Figure 6:**
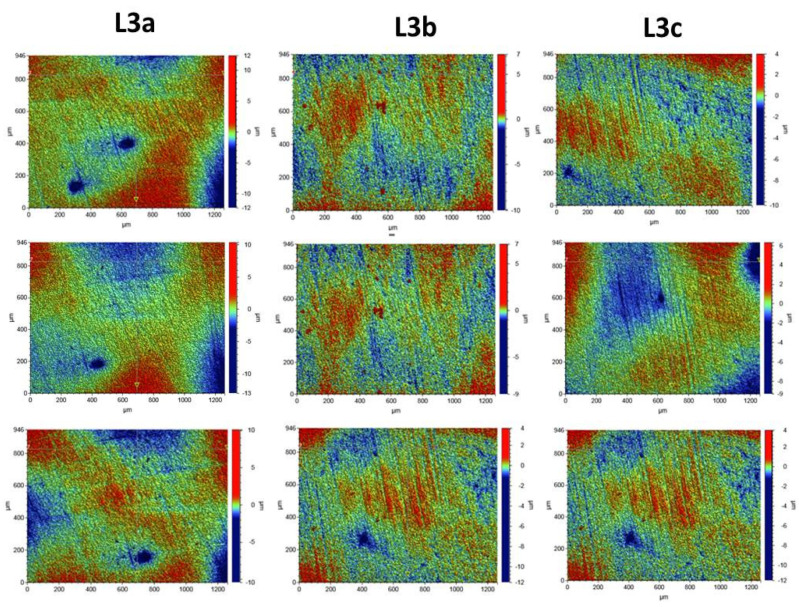
Surface microgeometry maps determined for the K2 composite for 3 variants of laser L3. L3a (periimplantitis variant, sweep technique, 60 s), L3b (periimplantitis variant, sweep technique, 2 × 30 s with 60 s cooling break) and L3c (periimplantitis variant, scoring technique, 60 s).

**Figure 7:**
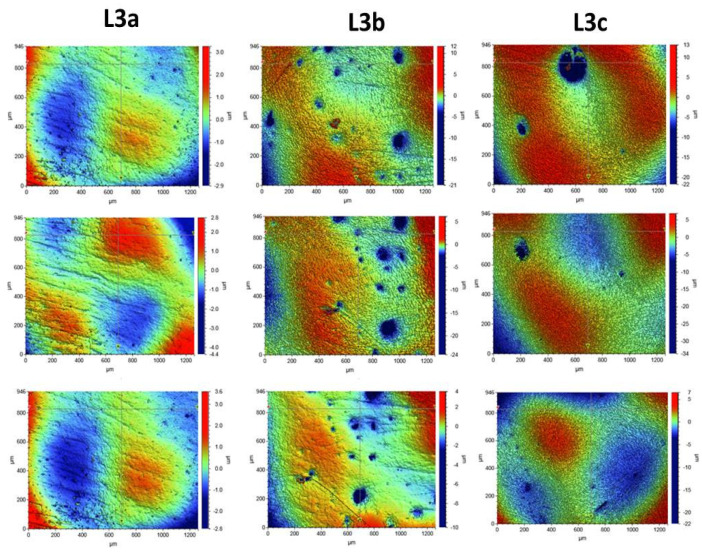
Surface microgeometry maps determined for the K3 composite for 3 variants of laser L3. L3a (periimplantitis variant, sweep technique, 60 s), L3b (periimplantitis variant, sweep technique, 2 × 30 s, with a 60 s cooling pause), and L3c (periimplantitis variant, point technique, 60 s).

Surface microgeometry maps were also made, and roughness parameters were determined for the surfaces polished with various polishing wheels and laser irradiated in three variants. For the surfaces exposed to laser irradiation, 3 measurements were made for each of the tested composites. The test results are shown in Figure 5, Figure 6 and Figure 7.

The tests showed that the roughness of the tested composites was similar. For all the tested samples before the laser irradiation process, the determined mean values of the roughness parameter Ra ranged from 0.578 to 0.696 µm. The K1 composite, polished with one polishing material—the Enhance polishing rubber (Dentsply Sirona)—exhibited the highest surface roughness. On its surface, local pits with the maximum depth of 15 µm were observed. The average Ra value for the K1 composite determined for the five examined areas was 0.696 ± 0.07 µm. The K2 composite, polished with two polishing materials, an eraser, and then a Sof-Lex™ Pre-Polishing Spiral (beige) disc, showed the smallest roughness. The average Ra value for the K2 composite determined for the five examined areas was 0.578 ± 0.06 µm. No local depressions were observed on the determined surface microgeometry maps. The average Ra value for the K3 composite polished with the use of three polishing materials, namely rubber, Sof-Lex™ Pre-Polishing Spiral (beige) disc, and final smoothing with the Sof-Lex™ Pre-Polishing Spiral (pink) polishing disc, was 0.61 ± 0.06 µm. On the microgeometry maps for the five selected areas, local pits with the maximum depth of 7 µm were observed.

The surface microgeometry maps made for the K1–K3 composites after the smoothing process and then lasering in three variants did not show any significant differences in the surface structure of the tested composites. As in the case of the composites, before the laser irradiation, the K1 composite had numerous depressions. Such pits are also visible on the microgeometry maps of the K3 composite surface after the laser process. In the case of K2 composite, the smallest number of depressions was observed on the surface microgeometry maps. No significant differences were found in the roughness parameters under the laser irradiation. The differences in these parameters are most likely due to the heterogeneity of the material and local waviness. The highest average value of the roughness parameter Ra after the laser process determined from three measurements was obtained for the K2 composite after the laser treatment with the L3b variant (2 × 30 s sweeping) and was 0.320 ± 0.03. The highest value of the Ra roughness parameter was obtained for the K3 composite in the L3c laser variant (60 s without any movements), with a value of 1.661 ± 0.16. No dependence of the Ra roughness parameter on the applied laser variant was observed. The research suggests that all variants of lasering can be used in the dental practice.

### 3.5. Evaluation of the Biocidal Effect of Laser Irradiation

The biocidal effect of a diode laser after irradiation in various variants is presented in Table 4.

The reduction in the number of microorganisms after the diode laser irradiation amounted to 38–100% depending on the species and the composite type. The greater the laser power used, the greater the reduction in the number of the tested bacteria and fungi. The smallest reduction in the number of microorganisms was demonstrated with the irradiation of the K1 composite with the L1 laser variant over 1 min. The L3 laser in both time variants proved to be the most effective against all tested species. The reduction ranged from 75 to 100%. The complete elimination of the microorganisms was achieved only for *Streptococcus pneumoniae* by irradiating the K2 and K3 composites with L3 laser over 1 min and with the L3 laser in the 2 × 30 s variant with a cooling break. In the case of the L3 laser, *Klebsiella oxytoca* was reduced the least effectively (70.75–87.89%). The largest percentage reduction of microorganisms was on the K2 composite. The microorganisms on the K1 composite were the most difficult to eradicate. In 10 out of 12 tested samples, the effectiveness of the L3 laser in the 2 × 30 s variant with a 1-min break for cooling proved to be more effective than irradiating with L3 for 1 min.

## 4. Discussion

Properly performed implant implantation procedure, systematic checks assessing the condition of tissues around implant, and maintaining proper oral hygiene by the patient minimize the risk of periimplantitis, i.e., loss of bone tissue around the implant. Hygiene is primarily aimed at reducing microbes, including pathogens that can be harmful to health. The nature of the contamination depends on the surrounding oral flora. In the case of high pollution, it most often leads to a persistent inflammatory reaction [19]. Many of the microorganisms identified on the composites and adjacent surfaces appear in the Human Oral Microbiome database as normal oral flora [26].

The largest contamination with microorganisms in relation to the surface of the zirconium crown, porcelain crown, and the tooth around the implant was found on the composites closing SAH. As in this paper, Cassio et al. [27] found medium and large numbers of pathogenic and non-pathogenic microbial species on the individual implant components. In this case, the composite also showed the largest microbial count.

Recently there has been a rapid development of metagenomic methods that allow for the detection of the presence of entire genomes of microorganisms in a sample in various environments [28,29]. These are the methods derived from the systemic methods of molecular biology and allow for the sequence analysis of the obtained genetic material. The sequences are electronically deposited in the databases in GenBank (Stanford, CA, USA), EMBL (European Molecular Biology Laboratory Nucleotide Sequence Database, UK), or DDBJ (DNA Data Bank of Japan, Mishima, Japan), which form one consortium—The International Sequence Database Collaboration—and exchange data on an ongoing basis.

The collected sequences are the subject of further analyses aimed at obtaining as much information as possible about a given genome and the functioning of the host cell. The levels of genome information analysis correspond to the steps in which this information is expressed in cells. Owing to such an advanced technique in these studies, similarly to those by Bor et al., non-cultivable microorganisms typical of the oral cavity—TM7 (Saccharibacteria)—were detected; however, the recent studies have revealed a very large diversity of 16S rRNA for this type of microorganism in mammals and their relationship with infectious diseases of the oral mucosa [30].

Optical profilometry and contact profilometry, which are often described in the literature, are very effective and commonly used methods of examining the surface of composites used in dentistry [31]. This method enables the registration of 3D images of the tested surface and the determination of metrological parameters. These parameters allow for a comprehensive assessment of the surface microgeometry of the tested materials. Surface microgeometries are usually described by the following roughness parameters: Ra, arithmetic mean of the elevation profile; Rq, the mean square elevation profile; Rt, the height of the highest peak profile; and Ry, the lowest recesses’ profile [21]. The roughness parameters of the composites used in dentistry depend, among others, on the material and its processing. The literature data clearly show that the use of polishing systems on the finished surface of the tested composite resins is clinically significant [32,33]. Therefore, the roughness parameters were examined depending on the polishing variants in this paper. The dependence of the roughness parameters of polished composites on the diode laser irradiation process in various variants was also investigated.

The tests carried out with the use of optical profilometry did not show very large differences in the roughness parameters of the tested composite depending on the polishing method. The smoothest surface was obtained for the K2 polished composite, using an eraser, followed by a Sof-Lex™ Pre-Polishing Spiral (beige) (3M™) polishing disc. However, the research showed a dependence of the percentage reduction of microorganisms present on the composites on their roughness. The highest percentage reduction of microorganisms was observed for the K2 composite, which is characterized by the smallest roughness. On the other hand, the smallest reduction of microorganisms was observed for the K1 composite, which was characterized by the largest surface roughness. The tests performed after the laser process also showed the smallest surface roughness of the K2 composite after the laser treatment with the L3b variant. Moreover, it was not observed that the L3a and L3c laser variants had a significant impact on the deterioration of the roughness parameters. The research showed clearly that the roughness parameters of the materials are important for the effective removal of microorganisms from their surfaces.

It is very important that dentists properly prepare the surface of the composite with which they close the SAH, because the greater the roughness of the surface, the more biofilms accumulate on it. The composite roughness depends on, among other things, the right polishing procedure. On the composites that are not smooth enough, in addition to biofilms, discoloration often appears that may be caused by the consumed food, as demonstrated also by Ji-Won Choi et al. [34] and is also presented in the figure in this paper.

According to the literature data, there is no threshold of unacceptable surface roughness of materials used in dentistry. Most of the published studies indicate that an Ra above 0.2 μm increases the accumulation of dental plaque and the risk of caries and periodontitis. This results in deterioration of the aesthetics and reduces the restoration durability [35,36]. Willems et al. [37] stated that the specific surface roughness of composite resins must be equal to or smaller than the roughness of human enamel on the enamel–enamel contact surfaces (Ra = 0.64 μm). The Ra value in this study was achieved for the composite polished with an eraser and a polishing wheel (beige).

In addition, the increase in roughness of the composites used in dentistry is also influenced by the material degradation depending on time and environmental factors. This is mainly due to the formation of biofilms. It was proved that the growth of *S. mutans* on the resin composite increases the surface roughness. Changing the surface integrity can further accelerate the biofilm accumulation [38]. Rinastiti also proved that the in vitro exposure of composites to oral biofilm causes clinically significant surface degradation. It has been concluded that in vitro exposure to the oral biofilm is the clinically significant state of aging [39].

It should be emphasized that the percentage of reduction of microorganisms from dental surfaces was analyzed. Thus, the initial number of microorganisms was not a determinant for the laser’s effectiveness. The effectiveness of the laser in reducing microorganisms from dental surfaces, depending on the surface roughness, may be related to the scattering and absorption of laser radiation on the surface.

In the case of roughnesses with a size close to and larger than the wavelength of the incident light, forward light scattering takes place—Mie scattering; meanwhile, roughnesses smaller than the wavelength of the radiation incident on them scatter the light backward—Rayleigh scattering. As a result of forward scattering (Mie scattering), the radiation beam leaves the matter moving in a direction deviating from the original by an angle of less than 90°. On the other hand, due to backscattering (Rayleigh scattering), the radiation travels backward from the original direction of motion by an angle greater than 90°. It should be noted that, the rougher the surface is, the greater the radiation scattering. One of the suggested mechanisms that can reduce the number of microorganisms is the increase in the surface temperature of the implant under the influence of laser light. Increased temperature may cause thermal degradation of the elements, thus allowing the adhesion of microorganisms to the substrate. The increased roughness of the substrate increases the radiation scattering relative to the absorption and, thus, may reduce the reduction factor of the number of microorganisms.

New methods are constantly being sought to limit the growth of microorganisms on the surfaces around the dental implants. The Elexxion Claros laser irradiation of the surfaces of composites inoculated with the most common microorganisms in the oral cavity environment in the periimplantitis surgical variants tested in this paper, i.e., 25W/15,000 Hz/10 µs, average = 3.84 W for 1 min, and 2 × 30 s gave, satisfactory results at the level of 75–100%. Similar results were obtained by Gutknecht et al. by laser-beam *Escherichia coli* and *Streptococcus faecalis* in the dental canal [40]. Comparable results were also achieved by Shanshan et al., who reduced *E. faecalis* by 94.94–99.44%, using a diode laser [41]. Medical lasers are used to decontaminate a wide variety of materials, and their effectiveness depends on the surface they are exposed to. The reduction of microorganisms was 92.17–100.00% on the cellulose material, and the reduction was 96–100% on the collagen ones. The more porous the structure, the smaller the reduction is [28,42].

Proper surface preparation by the dentist is very important in limiting the deposition of plaque on the composite surfaces. As demonstrated by van Noort and Davis, as well as van Dijken and Ruyter, the applied finishing and polishing procedures have an impact on the surface properties of the composite [43,44]. Different polishing sets can have different effects on the composite resin surfaces. It is possible to smooth the surface, but it can also be made rougher and, thus, increase the predisposition to the formation of biofilms, as confirmed by Carlen et al. [45].

The 3M™ ESPE™ Sof-Lex™ diamond polishing system is a two- or three-step multipurpose polishing system. The universal shape allows it to be used on all tooth surfaces. It reduces the need to use multi-shaped tools designed for specific shapes and contours. Such a system was selected for research because it is widely used and recommended by dentists. However, the intended results are not always achieved because, despite certain parameters, the method and technique of polishing depend mainly upon the dentist’s skills. Perhaps the solution to this problem would be the introduction of dental composites that would allow the dentist to visually verify whether the degree of polishing is sufficient to minimize the formation of biofilms. Taking into account the very fast development of nanomaterials, it seems that designing such a solution is possible.

## 5. Conclusions

The composites used to close the SAH are more microbiologically contaminated than the porcelain and zirconium used to build the implants. Proper preparation of the composite surface by polishing may limit the deposition of microorganisms, and the smoother the surface, the more effective biocidal treatment of the diode laser. The optimized doses of the diode laser do not show the destructive effect of the laser variants used on the surface of the tested composites.

## Figures and Tables

**Table 1:** Average number of microorganisms on the composites, porcelain, zirconium, and the teeth surface (detected by the culture dependent technique).

Patient	Teeth (CFU/0.4 cm^2^)	Porcelain (CFU/0.4 cm^2^)	Zirconium (CFU/0.4 cm^2^)	Composite (CFU/0.4 cm^2^)
1	4.50 × 10^6^ ± 0.36 × 10^6^	3.98 × 10^7^ ± 0.33 × 10^7^	1.10 × 10^8^ ± 0.30 × 10^8^	1.65 × 10^8^ ± 0.09 × 10^8^
2	1.44 × 10^4^ ± 0.61 × 10^4^	5.16 × 10^4^ ± 0.55 × 10^4^	2.47 × 10^5^ ± 0.60 × 10^5^	1.21 × 10^6^ ± 0.60 × 10^5^
3	3.58 × 10^3^ ± 0.45 × 10^3^	3.20 × 10^4^ ± 0.46 × 10^4^	1.16 × 10^6^ ± 0.54 × 10^6^	2.92 × 10^6^ ± 0.78 × 10^6^
4	1.60 × 10^6^ ± 2.77 × 10^6^	1.20 × 10^7^ ± 2.1 × 10^7^	4.50 × 10^7^ ± 7.6 × 10^7^	5.30 × 10^7^ ± 8.80 × 10^7^
5	1.30 × 10^6^ ± 2.30 × 10^6^	1.40 × 10^7^ ± 2.4 × 10^7^	4.10 × 10^7^ ± 6.9 × 10^7^	5.70 × 10^7^ ± 9.70 × 10^7^
6	1.50 × 10^6^ ± 2.6 × 10^6^	1.30 × 10^7^ ± 2.3 × 10^7^	2.50 × 10^7^ ± 4.3 × 10^7^	5.80 × 10^7^ ± 6.90 × 10^7^

Mean ± standard deviation.

**Table 2:** The average number of microorganisms identified using the MALDI–TOF MS technique on the surface of the composite, zirconium, porcelain, and tooth crown in Patients 1–6.

Microorganism	Patient	Teeth (CFU/0.4 cm^2^)	Porcelain (CFU/0.4 cm^2^)	Zirconium (CFU/0.4 cm^2^)	Composite (CFU/0.4 cm^2^)
*Candida* *guillermondi*	1	1.40 × 10^3^ ± 1.56 × 10^3^	1.50 × 10^5^ ± 0.96 × 10^5^	1.18 × 10^6^ ± 0.95 × 10^6^	6.00 × 10^7^ ± 0.79 × 10^7^
	2	1.00 × 10^3^ ± 0.70 × 10^3^	1.26 × 10^3^ ± 1.23 × 10^3^	1.38 × 10^3^ ± 1.07 × 10^3^	1.89 × 10^3^ ± 0.96 × 10^3^
	3	0.00 × 10^0^ ± 0.00 × 10^0^	1.67 × 10^2^ ± 1.42 × 10^2^	1.40 × 10^3^ ± 1.09 × 10^3^	3.20 × 10^4^ ± 0.73 × 10^4^
	4	0.00 × 10^0^ ± 0.00 × 10^0^	0.00 × 10^0^ ± 0.00 × 10^0^	0.00 × 10^0^ ± 0.00 × 10^0^	4.80 × 10^6^ ± 3.95 × 10^6^
	5	0.00 × 10^0^ ± 0.00 × 10^0^	0.00 × 10^0^ ± 0.00 × 10^0^	1.40 × 10^6^ ± 0.95 × 10^6^	3.08 × 10^6^ ± 1.85 × 10^6^
	6	0.00 × 10^0^ ± 0.00 × 10^0^	0.00 × 10^0^ ± 0.00 × 10^0^	3.20 × 10^6^ ± 1.95 × 10^6^	3.65 × 10^6^ ± 0.95 × 10^6^
*Klebsiella oxytoca*	1	0.00 × 10^0^ ± 0.00 × 10^0^	0.00 × 10^0^ ± 0.00 × 10^0^	0.00 × 10^0^ ± 0.00 × 10^0^	0.00 × 10^0^ ± 0.00 × 10^0^
	2	0.00 × 10^0^ ± 0.00 × 10^0^	0.00 × 10^0^ ± 0.00 × 10^0^	0.00 × 10^0^ ± 0.00 × 10^0^	0.00 × 10^0^ ± 0.00 × 10^0^
	3	1.20 × 10^3^ ± 1.40 × 10^2^	3.20 × 10^4^ ± 2.07 × 10^4^	6.13 × 10^5^ ± 3.24 × 10^5^	3.00 × 10^6^ ± 2.09 × 10^5^
	4	0.00 × 10^0^ ± 0.00 × 10^0^	0.00 × 10^0^ ± 0.00 × 10^0^	0.00 × 10^0^ ± 0.00 × 10^0^	0.00 × 10^0^ ± 0.00 × 10^0^
	5	2.20 × 10^1^ ± 0,30 × 10^1^	1.20 × 10^2^ ± 2,30 × 10^1^	4.30 × 10^2^ ± 2,30 × 10^1^	6.00 × 10^2^ ± 0.75 × 10^2^
	6	0.00 × 10^0^ ± 0.00 × 10^0^	0.00 × 10^0^ ± 0.00 × 10^0^	0.00 × 10^0^ ± 0.00 × 10^0^	0.00 × 10^0^ ± 0.00 × 10^0^
*Neisseria* *flavescens*	1	0.00 × 10^0^ ± 0.00 × 10^0^	0.00 × 10^0^ ± 0.00 × 10^0^	0.00 × 10^0^ ± 0.00 × 10^0^	0.00 × 10^0^ ± 0.00 × 10^0^
	2	0.00 × 10^0^ ± 0.00 × 10^0^	0.00 × 10^0^ ± 0.00 × 10^0^	1.00 × 10^2^ ± 1.54 × 10^2^	7.67 × 10^2^ ± 1.62 × 10^2^
	3	0.00 × 10^0^ ± 0.00 × 10^0^	0.00 × 10^0^ ± 0.00 × 10^0^	0.00 × 10^0^ ± 0.00 × 10^0^	0.00 × 10^0^ ± 0.00 × 10^0^
	4	0.00 × 10^0^ ± 0.00 × 10^0^	0.00 × 10^0^ ± 0.00 × 10^0^	0.00 × 10^0^ ± 0.00 × 10^0^	0.00 × 10^0^ ± 0.00 × 10^0^
	5	0.00 × 10^0^ ± 0.00 × 10^0^	0.00 × 10^0^ ± 0.00 × 10^0^	0.00 × 10^0^ ± 0.00 × 10^0^	0.00 × 10^0^ ± 0.00 × 10^0^
	6	0.00 × 10^0^ ± 0.00 × 10^0^	0.00 × 10^0^ ± 0.00 × 10^0^	0.00 × 10^0^ ± 0.00 × 10^0^	0.00 × 10^0^ ± 0.00 × 10^0^
*Neisseria perflava*	1	0.00 × 10^0^ ± 0.00 × 10^0^	0.00 × 10^0^ ± 0.00 × 10^0^	0.00 × 10^0^ ± 0.00 × 10^0^	0.00 × 10^0^ ± 0.00 × 10^0^
	2	0.00 × 10^0^ ± 0.00 × 10^0^	0.00 × 10^0^ ± 0.00 × 10^0^	0.00 × 10^0^ ± 0.00 × 10^0^	0.00 × 10^0^ ± 0.00 × 10^0^
	3	0.00 × 10^0^ ± 0.00 × 10^0^	0.00 × 10^0^ ± 0.00 × 10^0^	6.67 × 10^2^ ± 2.32 × 10^2^	4.33 × 10^2^ ± 2.71 × 10^2^
	4	0.00 × 10^0^ ± 0.00 × 10^0^	0.00 × 10^0^ ± 0.00 × 10^0^	0.00 × 10^0^ ± 0.00 × 10^0^	0.00 × 10^0^ ± 0.00 × 10^0^
	5	0.00 × 10^0^ ± 0.00 × 10^0^	0.00 × 10^0^ ± 0.00 × 10^0^	0.00 × 10^0^ ± 0.00 × 10^0^	0.00 × 10^0^ ± 0.00 × 10^0^
	6	0.00 × 10^0^ ± 0.00 × 10^0^	0.00 × 10^0^ ± 0.00 × 10^0^	0.00 × 10^0^ ± 0.00 × 10^0^	0.00 × 10^0^ ± 0.00 × 10^0^
*Neisseria subflava*	1	0.00 × 10^0^ ± 0.00 × 10^0^	3.00 × 10^6^ ± 2.91 × 10^6^	5.60 × 10^6^ ± 2.43 × 10^6^	1.40 × 10^6^ ± 1.35 × 10^6^
	2	0.00 × 10^0^ ± 0.00 × 10^0^	0.00 × 10^0^ ± 0.00 × 10^0^	0.00 × 10^0^ ± 0.00 × 10^0^	0.00 × 10^0^ ± 0.00 × 10^0^
	3	0.00 × 10^0^ ± 0.00 × 10^0^	0.00 × 10^0^ ± 0.00 × 10^0^	0.00 × 10^0^ ± 0.00 × 10^0^	0.00 × 10^0^ ± 0.00 × 10^0^
	4	3.84 × 10^3^ ± 2.00 × 10^3^	3.94 × 10^3^ ± 1.02 × 10^2^	2.04 × 10^4^ ± 3.02 × 10^2^	2.08 × 10^5^ ± 1.33 × 10^3^
	5	0.00 × 10^0^ ± 0.00 × 10^0^	0.00 × 10^0^ ± 0.00 × 10^0^	0.00 × 10^0^ ± 0.00 × 10^0^	0.00 × 10^0^ ± 0.00 × 10^0^
	6	0.00 × 10^0^ ± 0.00 × 10^0^	0.00 × 10^0^ ± 0.00 × 10^0^	0.00 × 10^0^ ± 0.00 × 10^0^	0.00 × 10^0^ ± 0.00 × 10^0^
*Rothia aeria*	1	2.00 × 10^5^ ± 1.64 × 10^5^	8.00 × 10^5^ ± 1.37 × 10^5^	2.00 × 10^6^ ± 1.23 × 10^6^	3.40 × 10^6^ ± 1.57 × 10^6^
	2	1.09 × 10^4^ ± 1.29 × 10^4^	1.83 × 10^4^ ± 1.01 × 10^4^	8.83 × 10^4^ ± 3.28 × 10^4^	5.45 × 10^5^ ± 3.30 × 10^5^
	3	0.00 × 10^0^ ± 0.00 × 10^0^	0.00 × 10^0^ ± 0.00 × 10^0^	0.00 × 10^0^ ± 0.00 × 10^0^	0.00 × 10^0^ ± 0.00 × 10^0^
	4	5.40 × 10^3^ ± 1.40 × 10^1^	1.30 × 10^4^ ± 2.70 × 10^4^	1.20 × 10^5^ ± 1.10 × 10^4^	6.20 × 10^5^ ± 1.30 × 10^5^
	5	0.00 × 10^0^ ± 0.00 × 10^0^	0.00 × 10^0^ ± 0.00 × 10^0^	6.30 × 10^3^ ± 1.70 × 10^4^	5.30 × 10^5^ ± 1.40 × 10^1^
	6	1.30 × 10^3^ ± 1.70 × 10^1^	3.60 × 10^3^ ± 2.40 × 10^2^	3.90 × 10^3^ ± 1.90 × 10^2^	0.00 × 10^0^ ± 0.00 × 10^0^
*Rothia* *dentocariosa*	1	1.20 × 10^6^ ± 0.91 × 10^6^	3.40 × 10^6^ ± 0.8 × 10^6^	9.00 × 10^6^ ± 5.92 × 10^6^	6.00 × 10^7^ ± 3.82 × 10^7^
	2	0.00 × 10^0^ ± 0.00 × 10^0^	0.00 × 10^0^ ± 0.00 × 10^0^	0.00 × 10^0^ ± 0.00 × 10^0^	0.00 × 10^0^ ± 0.00 × 10^0^
	3	1.43 × 10^3^ ± 1.11 × 10^3^	1.60 × 10^4^ ± 0.85 × 10^4^	6.13 × 10^5^ ± 4.67 × 10^5^	1.65 × 10^6^ ± 0.80 × 10^6^
	4	5.40 × 10^3^ ± 1.14 × 10^1^	1.20 × 10^4^ ± 1.05 × 10^4^	1.20 × 10^5^ ± 2.10 × 10^4^	1.60 × 10^6^ ± 3.50 × 10^4^
	5	2.20 × 10^2^ ± 1.30 × 10^1^	4.20 × 10^3^ ± 1.30 × 10^5^	6.20 × 10^5^ ± 1.30 × 10^5^	5.80 × 10^8^ ± 8.20 × 10^7^
	6	4.6 × 10^2^ ± 2.52 × 10^2^	5.6 × 10^3^ ± 2.52 × 10^2^	8.00 × 10^5^ ± 1.54 × 10^4^	2.10 × 10^6^ ± 5.09×10^4^
*Staphylococcus epidermidis*	1	0.00 × 10^0^ ± 0.00 × 10^0^	0.00 × 10^0^ ± 0.00 × 10^0^	0.00 × 10^0^ ± 0.00 × 10^0^	0.00 × 10^0^ ± 0.00 × 10^0^
	2	1.22 × 10^3^ ± 0.87 × 10^3^	2.67 × 10^2^ ± 1.29 × 10^2^	6.67 × 10^3^ ± 2.35 × 10^3^	1.00 × 10^4^ ± 0.72 × 10^4^
	3	1.67 × 10^2^ ± 1.94 × 10^2^	1.00 × 10^3^ ± 0.82 × 10^3^	1.33 × 10^4^ ± 0.68 × 10^4^	2.00 × 10^3^ ± 1.45 × 10^3^
	4	0.00 × 10^0^ ± 0.00 × 10^0^	0.00 × 10^0^ ± 0.00 × 10^0^	0.00 × 10^0^ ± 0.00 × 10^0^	0.00 × 10^0^ ± 0.00 × 10^0^
	5	0.00 × 10^0^ ± 0.00 × 10^0^	0.00 × 10^0^ ± 0.00 × 10^0^	0.00 × 10^0^ ± 0.00 × 10^0^	0.00 × 10^0^ ± 0.00 × 10^0^
	6	0.00 × 10^0^ ± 0.00 × 10^0^	0.00 × 10^0^ ± 0.00 × 10^0^	0.00 × 10^0^ ± 0.00 × 10^0^	0.00 × 10^0^ ± 0.00 × 10^0^
*Streptococcus parasanguinis*	1	0.00 × 10^0^ ± 0.00 × 10^0^	0.00 × 10^0^ ± 0.00 × 10^0^	0.00 × 10^0^ ± 0.00 × 10^0^	0.00 × 10^0^ ± 0.00 × 10^0^
	2	1.30 × 10^3^ ± 1.03 × 10^3^	3.20 × 10^4^ ± 1.50 × 10^4^	1.52 × 10^5^ ± 0.92 × 10^5^	5.53 × 10^5^ ± 1.30 × 10^5^
	3	1.88 × 10^3^ ± 0.81 × 10^3^	1.40 × 10^4^ ± 0.58 × 10^4^	4.33 × 10^5^ ± 1.24 × 10^5^	1.17 × 10^6^ ± 2.05 × 10^5^
	4	0.00 × 10^0^ ± 0.00 × 10^0^	0.00 × 10^0^ ± 0.00 × 10^0^	0.00 × 10^0^ ± 0.00 × 10^0^	0.00 × 10^0^ ± 0.00 × 10^0^
	5	0.00 × 10^0^ ± 0.00 × 10^0^	0.00 × 10^0^ ± 0.00 × 10^0^	0.00 × 10^0^ ± 0.00 × 10^0^	0.00 × 10^0^ ± 0.00 × 10^0^
	6	0.00 × 10^0^ ± 0.00 × 10^0^	0.00 × 10^0^ ± 0.00 × 10^0^	0.00 × 10^0^ ± 0.00 × 10^0^	0.00 × 10^0^ ± 0.00 × 10^0^
*Streptococcus pneumoniae*	1	3.00 × 10^6^ ± 2.02 × 10^6^	3.16 × 10^7^ ± 1.65 × 10^7^	8.36 × 10^7^ ± 1.10 × 10^7^	9.00 × 10^7^ ± 1.57 × 10^7^
	2	0.00 × 10^0^ ± 0.00 × 10^0^	0.00 × 10^0^ ± 0.00 × 10^0^	0.00 × 10^0^ ± 0.00 × 10^0^	0.00 × 10^0^ ± 0.00 × 10^0^
	3	0.00 × 10^0^ ± 0.00 × 10^0^	0.00 × 10^0^ ± 0.00 × 10^0^	0.00 × 10^0^ ± 0.00 × 10^0^	0.00 × 10^0^ ± 0.00 × 10^0^
	4	0.00 × 10^0^ ± 0.00 × 10^0^	0.00 × 10^0^ ± 0.00 × 10^0^	0.00 × 10^0^ ± 0.00 × 10^0^	0.00 × 10^0^ ± 0.00 × 10^0^
	5	1.30 × 10^3^ ± 2.07 × 10^3^	5.40 × 10^3^ ± 1.40 × 10^1^	9.10 × 10^4^ ± 6.60 × 10^3^	6.30 × 10^5^ ± 1.70 × 10^4^
	6	0.00 × 10^0^ ± 0.00 × 10^0^	0.00 × 10^0^ ± 0.00 × 10^0^	0.00 × 10^0^ ± 0.00 × 10^0^	0.00 × 10^0^ ± 0.00 × 10^0^

Mean ± standard deviation.

**Table 3:** Roughness parameters determined for the K1, K2, and K3 composites before and after the laser irradiation process in three variants: L3a (60 s sweeping), L3b (2 × 30 s sweeping), and L3c (60 s point technique).

Roughness Parameter Ra (µm)				
Measurement	K1	L3a	L3b	L3c
1	0.760 ± 0.07	1.041 ± 0.10	0.735 ± 0.07	0.840 ± 0.08
2	0.714 ± 0.07	1.052 ± 0.10	0.678 ± 0.07	1.044 ± 0.10
3	0.665 ± 0.06	1.354 ± 0.13	0.646 ± 0.06	0.903 ± 0.09
4	0.757 ± 0.07	-	-	-
5	0.584 ± 0.06	-	-	-
Average value	0.696 ± 0.07	1.149 ± 0.11	0.686 ± 0.07	0.930 ± 0.09
Measurement	K2	L3a	L3b	L3c
1	0.486 ± 0.05	0.664 ± 0.06	0.325 ± 0.03	0.359 ± 0.03
2	0.724 ± 0.07	0.850 ± 0.08	0.306 ± 0.03	0.646 ± 0.06
3	0.516 ± 0.05	0.544 ± 0.05	0.330 ± 0.03	0.330 ± 0.03
4	0.532 ± 0.05	-	-	-
5	0.632 ± 0.06	-	-	-
Average value	0.578 ±0.06	0.686 ± 0.07	0.320 ± 0.03	0.445 ± 0.04
Measurement	K3	L3a	L3b	L3c
1	1.080 ± 0.10	0.452 ± 0.04	0.982 ± 0.09	1.879 ± 0.18
2	0.576 ± 0.06	0.563 ± 0.05	0.658 ± 0.06	1.860 ± 0.18
3	0.591 ± 0.06	0.563 ± 0.05	0.662 ± 0.06	1.246 ± 0.12
4	0.481 ± 0.05	-	-	-
5	0.322 ± 0.03	-	-	-
Average value	0.610 ± 0.06	0.526 ± 0.05	0.767 ± 0.07	1.661 ± 0.16

Mean ± uncertainty.

**Table 4:** The results of the effectiveness of laser irradiation against the most common and pathogen microorganisms on the composites.

Microorganism	Type of Sample					
	Composite	Time of Exposure				
		Unirradiated	L1 (1 min)	L2 (1 min)	L3 (1 min)	L3 (2 × 30 s)
		Average Number of Microorganisms (CFU/0.4 cm^2^)	Reduction (%)			
*Candida guillermondi*	K1	5.05 × 10^7^ ± 2.25 × 10^7^	41.09 *	57.38 *	75.91 *	83.06 *
	K2		69.77 *	75.45 *	93.61 *	94.92 *
	K3		53.66 *	73.27 *	86.74 *	94.16 *
*Klebsiella oxytoca*	K1	8.91 × 10^5^ ± 3.17 × 10^5^	38.38 *	62.66 *	70.75 *	74.35 *
	K2		55.28 *	78.66 *	87.83 *	87.89 *
	K3		47.22 *	78.68 *	75.48 *	75.94 *
*Rothia dentocariosa*	K1	2.33 × 10^5^ ± 6.54 × 10^5^	40.75 *	58.04 *	98.36 *	99.28 *
	K2		44.41 *	74.35 *	87.89 *	99.97 *
	K3		41.55 *	64.66 *	99.28 *	99.41 *
*Streptococcus* *pneumoniae*	K1	5.15 × 10^7^ ± 2.15 × 10^7^	42.96 *	80.45 *	96.87 *	99.00 *
	K2		67.44 *	85.26 *	100.00 *	99.90 *
	K3		58.34 *	82.77 *	100.00 *	99.22 *

* Statistically significant difference in reference to the control sample; ANOVA and LSD at the significance level *p* < 0.05. L1—decontaminate implant: 1 W/CW, mean = 1.0 W, tip 600 μm, t = 1 min. L2—expose implant: 15 W/15,000 Hz/10 μs, mean = 2.30 W, tip 600 μm, t = 1 min. L3—periimplantitis surgical: 25 W/15,000 Hz/10 μs, average = 3.84 W, tip 600 μm, t = 1 min and t = 2 × 30 s.
